# Risk factors for quinolone-resistant *Klebsiella pneumoniae* infection in hospitalized children

**DOI:** 10.3389/fped.2026.1775720

**Published:** 2026-03-20

**Authors:** Xiao-Qian Xu, Man-Man Cai, Xiao-Yan Zhang, Chen-Chen Tian, He-Ping Cai

**Affiliations:** 1 Department of Clinical Pharmacy, Anhui Provincial Children’s Hospital, Hefei, Anhui, China; 2 Department of Pharmacy, Jiading District Central Hospital, Shanghai University of Medicine & Healthy Science, Shanghai, China

**Keywords:** children, ICU admission, quinolone-resistant *Klebsiella pneumoniae*, risk factors, sputum suction

## Abstract

**Objective:**

This study aimed to investigate the risk factors associated with quinolone-resistant *Klebsiella pneumoniae* (QRKP) infection in children hospitalized and to provide evidence for the prevention and management of this bacterial infection.

**Methods:**

A retrospective analysis was conducted on the clinical data of 274 children with QRKP infection admitted to Anhui Provincial Children's Hospital between January 1, 2020, and November 22, 2022. These children were divided into two groups: the drug-resistant (120 cases) and -sensitive group (154 cases). Univariate analysis was performed to identify factors with statistically significant differences between the two groups, followed by logistic regression analysis to identify independent risk factors for quinolone resistance.

**Results:**

Univariate analysis revealed that the drug-resistant group had a significantly higher proportion of children with a history of hospitalization, ICU admission, immunosuppressant use, antibiotic use, mechanical ventilation, and sputum suction compared to the sensitive group (all *P* < 0.05). Multivariate logistic regression analysis identified two independent risk factors for QRKP infection in children: a history of ICU admission within the past three months (*OR* = 4.5361, *P* = 1.35e-06) and sputum suction during the current hospitalization (*OR* = 2.3677, *P* = 0.0019).

**Conclusion:**

A history of ICU admission in the past three months and sputum suction during hospitalization are major risk factors for QRKP infection in children. Clinicians should implement routine QRKP screening upon admission and strengthen infection control measures to reduce the incidence of this infection in pediatric patients.

## 1 Introduction

*Klebsiella pneumoniae* (KP) infection is a prevalent condition in pediatric populations, often affecting the respiratory tract, nervous system, and surgical sites, leading to severe clinical manifestations such as pneumonia, sepsis, meningitis, and liver abscess (1, 2). In critical cases, KP infection can result in multiple organ failure and, in some instances, death (3). Quinolones, a class of synthetic antibiotics that target bacterial type II topoisomerases to exert antibacterial effects, are routinely used to treat infections caused by multidrug-resistant organisms and have been developed into third- and fourth-generation formulations, proving effective in clinical settings (4). However, the emergence of quinolone-resistant *Klebsiella pneumoniae* (QRKP) has raised significant concerns regarding treatment efficacy. High-risk factors, including hospital-based antibiotic usage and prolonged exposure, may drive the development of resistance mechanisms, leading to a reduction in the clinical efficacy of quinolones. Therefore, understanding the key risk factors for QRKP infection is crucial for devising effective prevention and management strategies in clinical practice.

Pediatric patients, particularly those under 3 years of age, are at increased risk for KP infection due to their immature immune systems and limited immune defenses (5, 6). In addition, the available antibacterial options for children are often limited due to the toxicity of certain drugs, posing challenges for treatment. While QRKP infections are well-documented in adult populations, there is a relative paucity of studies examining the risk factors for QRKP in children. Most domestic studies have been focused on large general hospitals treating adult patients, leaving a gap in our understanding of pediatric QRKP infection dynamics. This study aimed to investigate the risk factors for quinolone resistance among pediatric patients with KP infections, in order to provide evidence for improving the prevention and control of QRKP infections in pediatric settings.

## 2 Methods

### 2.1 Study design

This retrospective study was conducted at Anhui Provincial Children's Hospital using data extracted from the hospital's electronic medical record system. Collected variables included demographic characteristics, admission departments, underlying respiratory diseases, hospitalization-related exposures within the previous three months, and exposures during the current hospitalization. These variables were analyzed to identify risk factors associated with resistance to quinolone antibiotics (ciprofloxacin and levofloxacin) and QRKP infection in children.

The study was approved by the Medical Ethics Committee of Anhui Provincial Children's Hospital (approval No. 2022_YW_001). The requirement for informed consent was waived due to the retrospective design.

### 2.2 Study participants

A total of 274 children with KP infection hospitalized at our institution between January 1, 2020, and November 22, 2022, were consecutively enrolled. Based on quinolone susceptibility results, patients were classified into a drug-resistant group (120 cases) and a -sensitive group (154 cases). All clinical specimens were collected and processed in accordance with the National Code of Practice for Clinical Examination. Antimicrobial susceptibility testing was performed using the Kirby–Bauer disk diffusion method, and results were interpreted according to the Clinical and Laboratory Standards Institute (CLSI) guidelines (M100-S24, 2014) (7).

Inclusion criteria: all cases meet the above diagnostic requirements; hospitalization time > 2 days; age ≤ 14 years old; complete clinical data. Exclusion criteria: combined with other pathogenic bacteria infection cases; participants with severe liver and kidney insufficiency, etc. Each participant was included only once in the present study.

### 2.3 Data collection

The following variables were collected: (1) Demographic characteristics: age, sex. (2) Admission and treatment departments: categorized into the Pediatric Intensive Care Unit (PICU), pediatric internal medicine, pediatric general surgery, pediatric hematology and oncology, and the neonatology department. (3) Respiratory system comorbidities: presence of any underlying respiratory diseases. (4) Hospitalization-related exposures within the past 3 months: history of hospitalization, ICU admission, immunosuppressant use, and antibiotic use. (5) Current hospitalization exposures: central venous catheterization, tracheotomy/mechanical ventilation, surgery, and sputum suction.

### 2.4 Statistical analysis

Statistical analyses were performed using R software (version 4.1.0). Continuous variables with non-normal distributions are presented as medians with interquartile ranges (IQRs, 25th–75th percentiles), while categorical variables are expressed as counts and percentages. The Wilcoxon rank-sum test was used to compare age between the two groups. Categorical variables, including sex, admission department, presence of underlying respiratory diseases, hospitalization-related exposures within the previous three months, and exposures during the current hospitalization, were compared using the chi-square test.

For regression analyses, quinolone susceptibility status was coded as 0 for the drug-sensitive group and 1 for -resistant group. Univariate logistic regression models were constructed using the “glm” function in R to estimate odds ratios (*OR*s) with 95% confidence intervals (*CI*s) and corresponding *P* values. Variables with statistical significance in univariate analysis (*P* < 0.001) were included in the multivariate logistic regression model. Forest plots of the univariate analysis results were generated using the “forestplot” R package. Multivariate logistic regression analysis was subsequently performed to identify independent risk factors for quinolone-resistant *Klebsiella pneumoniae* infection.

## 3 Results

### 3.1 Univariate analysis results

Univariate analysis showed that age, sex, admission department, and the presence of underlying respiratory diseases were not significantly associated with quinolone resistance in children (all *P* > 0.05). In contrast, analyses of hospitalization-related exposures revealed that children in the drug-resistant group had significantly higher proportions of prior hospitalization, ICU admission, immunosuppressant use, and antibiotic use within the preceding three months compared with those in the quinolone-sensitive group (*P* = 4.14e-07, 0.0025, and 1.36e-05, 2.81e-05, respectively). Furthermore, mechanical ventilation and sputum suction during the current hospitalization were also significantly associated with an increased risk of quinolone resistance (*P* = 0.0013 and 5.08e-11, respectively). Overall, these findings indicate that hospitalization-related exposures within the previous three months, as well as mechanical ventilation and sputum suction during the current hospitalization, are important risk factors for quinolone resistance in pediatric patients (Table 1).

**Table 1:** Univariate analysis of the influence on the occurrence of qRKP infection in children.

Variables	Drug-sensitive group (*n* = 154)	Drug-resistant group (*n* = 120)	*W*/*χ*^2^	*P*
Age (months)	7.79 (2.07, 60.00)	5.55 (1.47, 34.50)	10,000	0.2431
Gender			0.0692	1
Males (*n* = 154)	87 (56.49%)	67 (55.83%)		
Females (*n* = 120)	67 (43.51%)	53 (44.17%)		
Admission department			8.7637	0.0673
PICU (*n* = 56)	27 (17.53%)	29 (24.17%)		
Pediatric internal medicine (*n* = 69)	43 (27.92%)	26 (21.67%)		
Pediatric general surgery (*n* = 67)	42 (27.27%)	25 (20.83%)		
Pediatric hematology oncology (*n* = 34)	22 (14.29%)	12 (10.00%)		
Neonatology (*n* = 48)	20 (12.99%)	28 (23.33%)		
History of underlying respiratory disease			0.28669	0.5923
Yes (*n* = 19)	9 (5.84%)	10 (8.33%)		
No (*n* = 253)	134 (92.86%)	110 (91.67%)		
Unknown (*n* = 2)	2 (0.48%)	0 (0.00%)		
History of hospitalization within the past three months			25.6290	4.14e-07
Yes (*n* = 181)	82 (45.45%)	99 (82.50%)		
No (*n* = 89)	70 (53.25%)	19 (15.83%)		
Unknown (*n* = 4)	2 (1.30%)	2 (1.67%)		
ICU history in the past three months			9.1291	0.0025
Yes (*n* = 60)	23 (14.94%)	37 (30.83%)		
No (*n* = 212)	130 (84.42%)	82 (68.33%)		
Unknown (*n* = 2)	1 (0.64%)	1 (0.84%)		
History of immunosuppressant use within the past three months			18.9210	1.36e-05
Yes (*n* = 81)	38 (24.68%)	43 (35.83%)		
No (*n* = 90)	72 (46.75%)	18 (15.00%)		
Unknown (*n* = 103)	44 (28.57%)	59 (49.17%)		
History of antibiotic use within the past three months			17.5410	2.81e-05
Yes (*n* = 174)	83 (53.90%)	91 (85.83%)		
No (*n* = 72)	56 (36.36%)	16 (13.33%)		
Unknown (*n* = 28)	15 (9.74%)	13 (10.83%)		
Central venous catheterization during this hospitalization			3.3633	0.1860
Yes (*n* = 80)	38 (24.68%)	42 (35.00%)		
No (*n* = 186)	110 (71.42%)	76 (63.33%)		
Tested positive at initial (*n* = 6)	4 (2.60%)	2 (1.67%)		
Unknown (*n* = 2)	2 (1.30%)	0 (0.00%)		
Mechanical ventilation during this hospitalization			11.5040	0.0013
Yes (*n* = 71)	22 (17.53%)	44 (36.66%)		
No (*n* = 192)	75 (76.62%)	74 (61.67%)		
Tested positive at initial (*n* = 9)	7 (4.55%)	2 (1.67%)		
Unknown (*n* = 2)	2 (1.30%)	0 (0.00%)		
Surgery during this hospitalization			0.1406	0.9321
Yes (*n* = 64)	37 (24.03%)	27 (22.50%)		
No (*n* = 176)	97 (62.98%)	79 (65.83%)		
Tested positive at initial (*n* = 32)	18 (11.69%)	14 (11.67%)		
Unknown (*n* = 2)	2 (1.30%)	0 (0.00%)		
Sputum suction during this hospitalization			47.4060	5.08e-11
Yes (*n* = 104)	43 (27.92%)	61 (50.83%)		
No (*n* = 160)	101 (65.58%)	59 (49.17%)		
Tested positive at initial (*n* = 9)	9 (5.85%)	0 (0.00%)		
Unknown (*n* = 1)	1 (0.65%)	0 (0.00%)		

### 3.2 Multivariate analysis results

Based on the above findings, univariate logistic regression analyses were performed for the identified variables. The results demonstrated that hospitalization within the previous three months, ICU admission within the previous three months, use of immunosuppressants within the previous three months, antibiotic use within the previous three months, mechanical ventilation during the current hospitalization, and sputum suction during the current hospitalization were all significantly associated with quinolone resistance in children (*OR* = 4.4480, 2.5504, 4.5263, 3.8373, 2.5435, 2.7864, all *P* < 0.05) (Table 2).

**Table 2:** Univariate logistic regression analysis of quinolone antibiotic resistance.

Variables	*OR*	95%*CI*	*P*
History of hospitalization within the past three months	4.4480	2.5183–8.1596	5.83e-07
ICU history in the past three months	2.5504	1.4244–4.6488	0.0018
History of immunosuppressant use within the past three months	4.5263	2.3348–9.0698	1.20e-05
History of antibiotic use within the past three months	3.8373	2.0833–7.3953	2.89e-05
Mechanical ventilation during this hospitalization	2.5435	1.5349–4.3054	0.0004
Sputum suction during this hospitalization	2.7864	1.7502–4.5125	2.16e-05

Subsequently, a multivariate logistic regression model was subsequently constructed including ICU admission within the previous three months and sputum suction during the current hospitalization, which showed the strongest associations in the univariate analysis. Variables such as prior hospitalization, immunosuppressant use, and antibiotic use were excluded from the multivariate model because of substantial missing data. The multivariate analysis indicated that ICU admission within the past three months and sputum suction during the current hospitalization remained significantly associated with quinolone resistance in children (*OR* = 4.5361, 95% *CI*: 2.4982–8.5637, *P* = 1.35e-06; *OR* = 2.3677, 95% *CI*: 1.3782–4.1096, *P* = 0.0019, respectively) (Table 3).

**Table 3:** Multivariate logistic regression analysis.

Variables	*OR*	95%*CI*	*P*
ICU history in the past three months	4.5361	2.4982–8.5637	1.35e-06
Sputum suction during this hospitalization	2.3677	1.3782–4.1096	0.0019

## 4 Discussion

Quinolones are a class of synthetic antibacterial agents that exert their antimicrobial effects primarily by inhibiting bacterial DNA replication and transcription through interference with bacterial type II topoisomerases. These agents are predominantly active against Gram-negative bacteria and demonstrate relatively weaker activity against Gram-positive organisms. Notably, despite their restricted use in pediatric populations, high resistance rates to quinolones among Gram-negative bacilli have been reported. For example, a previous study reported that resistance to both ciprofloxacin and levofloxacin was observed in 62.8% (27/43) of KP isolates (8). Pneumonia caused by drug-resistant bacterial infections in children remains a major clinical challenge, often leading to prolonged hospitalization and adversely affecting treatment outcomes and prognosis. Therefore, timely identification of factors associated with QRKP infection in pediatric patients is essential. A better understanding of these risk factors may facilitate the implementation of targeted preventive strategies and contribute to improved clinical outcomes in this population.

This study comprehensively evaluated the risk factors associated with QRKP infection in hospitalized children. The findings indicate that hospitalization, immunosuppressant use, and antibiotic exposure within the previous three months, as well as sputum suction, ICU admission, and mechanical ventilation during hospitalization, were significantly associated with reduced susceptibility of K. pneumoniae to quinolone antibiotics in pediatric patients. Furthermore, multivariate logistic regression analysis focusing on the most strongly associated variables demonstrated that ICU admission within the past three months and sputum suction during the current hospitalization remained independently associated with quinolone resistance in children.

KP is widely distributed in the hospital environment, and children, as a vulnerable population, have immature immune systems and limited resistance to external pathogenic microorganisms. Exposure to the hospital setting therefore increases the risk of KP infection or colonization (9). Consequently, children with a history of hospitalization within the previous three months should be considered a high-risk group for KP infection. Reducing unnecessary hospitalization duration and strengthening environmental disinfection during hospitalization are essential measures to ensure patient safety.

Previous studies have shown that the use of immunosuppressants and exposure to multiple antimicrobial agents are independent risk factors for reduced antimicrobial susceptibility of KP (10–12). In addition, immunosuppressive therapy impairs host immune function, thereby decreasing resistance to infection and potentially inducing or exacerbating infectious processes (13). Similarly, prolonged or inappropriate antibiotic use promotes the selective survival of drug-resistant bacteria, which may gradually become dominant strains. Numerous reports have documented antibiotic-resistant infections in pediatric populations (14, 15). In contrast, standardized and rational antibiotic use represents a protective factor against infection in children, and selecting appropriate antimicrobial agents based on microbiological susceptibility testing may reduce infection rates to some extent.

The intensive care unit (ICU) is widely recognized as a high-risk setting for KP infection (16). This may be attributed to prolonged hospital stays, severe disease conditions, and the presence of significant underlying comorbidities among ICU patients. Children admitted to the ICU often exhibit compromised immune function and reduced resistance to infection, which increases their susceptibility to KP.

Invasive procedures, such as mechanical ventilation and sputum suction, may cause varying degrees of mechanical injury and disrupt the integrity of the respiratory barrier, thereby facilitating the invasion of exogenous pathogens. Moreover, biofilms readily form on the inner surfaces of indwelling medical devices, making bacteria difficult to eradicate with antibiotics and promoting the development of antimicrobial resistance. These mechanisms may ultimately contribute to multidrug-resistant infections in children with pneumonia (17). In the present study, mechanical ventilation and sputum suction during hospitalization were significantly associated with QRKP infection. Therefore, minimizing invasive procedures, shortening their duration, and reinforcing standardized operational practices among medical staff may effectively reduce the risk of QRKP infection. In addition, previous document reported by Naryzhny et al. has identified medical equipment as an important route for cross-transmission of KP (18), highlighting the need for strict disinfection and sterilization protocols in hospital settings. The lack of data of molecular microbiological analysis that confirms bacterial resistance to quinolones is a limitation of this study. Additionally, the single-center retrospective design conducted in a pediatric population from one hospital represents another limitation, which may restrict the generalizability of our findings to other institutions or regions.

The types of antibacterial drugs available to children are limited, and clinicians should comprehensively analyze all aspects of information and materials in the treatment of infections caused by KP, and combine drugs reasonably. Active screening at admission allows early intervention. According to the children's clinical characteristics and drug sensitivity results, make a correct diagnosis and weigh the pros and cons to choose the appropriate treatment plan, so as to achieve the best therapeutic effect.

## Data Availability

The raw data supporting the conclusions of this article will be made available by the authors, without undue reservation.
